# Serum uric acid level as a cardio-cerebrovascular event risk factor in middle-aged and non-obese Chinese men

**DOI:** 10.18632/oncotarget.15902

**Published:** 2017-03-04

**Authors:** Zhi-Jun Li, Chen-Ju Yi, Jing Li, Na Tang

**Affiliations:** ^1^ Department of Neurology, Tongji Hospital, Tongji Medical College, Huazhong University of Science and Technology, Wuhan, China

**Keywords:** serum uric acid, cardio-cerebrovascular events, middle-age, non-obese, Gerotarget

## Abstract

**Aim:**

The role of uric acid as a risk factor for cardio-cerebrovascular diseases is controversial. In this study, we aimed to investigate the relationship between serum uric acid level and the risk of cardio-cerebrovascular events in middle-aged and non-obese Chinese men.

**Methods:**

We included 3152 participants from the health examination center of Tongji Hospital from June 2007 to June 2010. Clinical examination and medical records were collected at the annual health examination. The hazard ratios (HRs) of uric acid for cardio-cerebrovascular events were calculated by Cox proportional hazards models. Generalized additive model and threshold effect analysis were used to explore the non-linear relationship between serum uric acid level and the incidence of cardio-cerebrovascular event.

**Result:**

The mean follow-up time was 52 months. When the participants were classified into four groups by the serum acid quarter (Q1-Q4), the HRs (95% CI) of Q2-Q4 for cardio-cerebrovascular events were 1.26 (0.83, 1.92), 1.97 (1.33, 2.91) and 2.05 (1.40, 3.01), respectively, compared with the reference (Q1). The actual incidence and conditional incidence of cardio-cerebrovascular events in the high serum acid group were higher than those in the low serum acid group, which were stratified by the turning point (sUA = 372 mol/L). We also showed a strong prognostic accuracy of the multiple variable-based score in 3 years and 5 years, with area under the receiver operating characteristic (ROC) curve of 0.790 (0.756-0.823) and 0.777 (0.749-0.804), respectively.

**Conclusion:**

Serum uric acid level is a strong risk factor for cardio-cerebrovascular events.

## INTRODUCTION

Cardiovascular and cerebrovascular diseases, including coronary artery disease, acute myocardial infarction, stroke, and so on, are well known to be the most common causes of morbidity and mortality worldwide [1, 2]. Biological/life style factors, including aging, smoking, hyperlipidemia, hypertension, and obesity are well known risk factors for cardio-cerebrovascular diseases (CCBVDs) [3–5]. Recent studies also have called attention to another perspective on hyperuricemia, a product of purine metabolism in humans wherein excess accumulation can lead to various diseases and would be a significant predictor for the CCBVD development [6–8]. A study showed that high serum uric acid (sUA) levels were associated with risk of myocardial infarction and stroke during a mean follow-up of 8.4 years [9]. Odden et al. recently included 10,956 participants from the National Health and Nutrition Examination Survey and demonstrated an association between uric acid level and cardiovascular mortality even after adjustment for potential confounders [10]. However, some studies only found an association in women, and the associations disappeared after adjustment for confounders in others [11–13].

Because the serum uric acid level determination is widely available and inexpensive, a better understanding of its role as a risk factor is certainly warranted. In this study, we investigated the association between serum uric acid level and cardiovascular and cerebrovascular events (CCBVEs) in a large population-based longitudinal cohort in generally middle-aged and non-obese Chinese men who were CCBVD-free at baseline.

## RESULTS

### Subject characteristics

The mean follow-up period was 52 months (range, 12-71 months). Of the 3152 total participants, 285 (9.04%) had CCBVEs during follow-up. The baseline characteristics of the 3152 patients stratified by cardio-cerebrovascular events are shown in Table 1. The mean age was 51.1±5.4 years and 48.7±5.8 years in participants with CCBVEs and non-CCBVEs, respectively (*P* < 0.001). Higher body mass index (BMI), systolic blood pressure (SBP), and diastolic blood pressure (DBP) were observed in participants with CCBVEs, as well as drinking and smoking. The concentration of sUA was 328.1±74.3 μmol/L in participants with non-CCBVEs, whereas participants with CCBVEs showed a higher sUA concentration of 359.4±78.6 μmol/L. Albumin, total bilirubin, glucose, blood urea nitrogen (BUN), creatinine, sUA, total cholesterol (TC), triglyceride (TG), low-density lipoprotein cholesterol (LDL-C), and white blood cell (WBC) were significantly higher in participants with CCBVEs, whereas high-density lipoprotein cholesterol (HDL-C) was lower.

**Table 1:** Baseline characteristics of subjects

Cardio-cerebrovascular Events	Non (*N*= 2867)	Yes (*N*= 285)	*P*-value
**Clinical parameters**			
Age, year	48.7 ± 5.8	51.1 ± 5.4	0.943
BMI, kg/m^2^	22.3 ± 1.8	23.3 ± 1.4	<0.001
Drinking	430 (15.0%)	80 (28.1%)	<0.001
Smkoing	285 ( 9.9%)	63 (22.1%)	<0.001
Hypertension	645 (22.5%)	96 (33.7%)	<0.001
SBP, mmHg	126.6 ± 15.1	132.1 ± 15.4	<0.001
DBP, mmHg	78.6 ± 10.5	83.3 ± 10.6	<0.001
**Laboratory parameters**			
Albumin, g/L	44.6 ± 2.5	44.8 ± 2.5	0.104
Total bilirubin,	13.1 ± 5.1	12.3 ± 4.0	0.013
BUN, mmol/L	4.9 ± 1.2	4.8 ± 1.2	0.137
Creatinine, μmol/L	89.1 ± 16.3	91.5 ± 14.7	0.016
Ccr, ml/min	82.4 ± 16.2	84.1 ± 16.1	0.083
Uric acid, μmol/L	328.1 ± 74.3	359.4 ± 78.6	<0.001
Glucose, mmol/L	5.4 ± 1.1	5.6 ± 1.1	0.003
Total cholesterol, mmol/L	5.0 ± 0.9	5.2 ± 1.1	0.001
Triglyceride, mmol/L	1.7 ± 1.2	2.4 ± 2.4	<0.001
HDL-C, mmol/L	1.4 ± 0.3	1.3 ± 0.3	<0.001
LDL-C, mmol/L	2.5 ± 0.6	2.6 ± 0.6	0.132
Hemoglobin, g/L	147.9 ± 9.8	148.8 ± 9.1	0.114
Platelet count	189.0 ± 48.4	189.7 ± 46.7	0.793
White blood cell	6.1 ± 1.6	6.6 ± 1.6	<0.001

BMI, body mass index; SBP, systolic blood pressure; DBP, diastolic blood pressure; BUN, blood urea nitrogen; Ccr, Creatinine clearance rate; HDL-C, high-density lipoprotein cholesterol; LDL-C, low-density lipoprotein cholesterol.

### Higher sUA level is related to higher prevalence of CCBVEs

The participants were classified into four groups by the sUA quarter, Q1 < 279 μmol/L, 279 μmol/L ≤ Q2 < 323.5 μmol/L, 323.5μmol/L ≤ Q3 < 375 μmol/L, and Q4 ≥ 375 μmol/L, to derive a deeper understanding of the relationship between sUA level and risk of CCBVEs. The incidence of CCBVEs was 4.9%, 6.6%, 10.4%, and 14.4% in four groups, respectively. Numbers of each event in different sUA groups were shown in Supplementary Table 1. Subsequently, Cox proportional hazard regression was performed to detect the hazard ratios (HRs) of sUA for CCBVEs. The HRs for incidence of CCBVEs substantially increased with increasing concentrations of sUA. Considering Q1 as the reference group, Table 2 shows that Q2, Q3, and Q4 had higher risks for CCBVEs, with HRs of 1.42 (0.94, 2.15), 2.21 (1.51, 3.24), and 3.03 (2.10, 4.36), respectively. When adjusted for clinical parameters such as smoking, drinking, age, BMI, SBP, and DBP, the HRs of Q2-Q4 were 1.23 (0.82, 1.87), 2.04 (1.39, 3.00), and 2.39 (1.66, 3.45), respectively. The HRs from adjusted II were further decreased after further adjusting for other known confounding variables. Compared with the reference, the HRs of Q2-Q4 were 1.26 (0.83, 1.92), 1.97 (1.33, 2.91), and 2.05 (1.40, 3.01), respectively. In addition, we investigated the interaction between age, hypertension, creatinine clearance rate (CCr), glucose, TG, and sUA for risk of CCBVEs. Table 3 shows that participants with higher age, CCr, glucose, TG, and hypertension had lower HRs of sUA. However, we did not observe a significant interaction between the five confounding variables and sUA (all P for interaction > 0.05).

**Figure 1:**
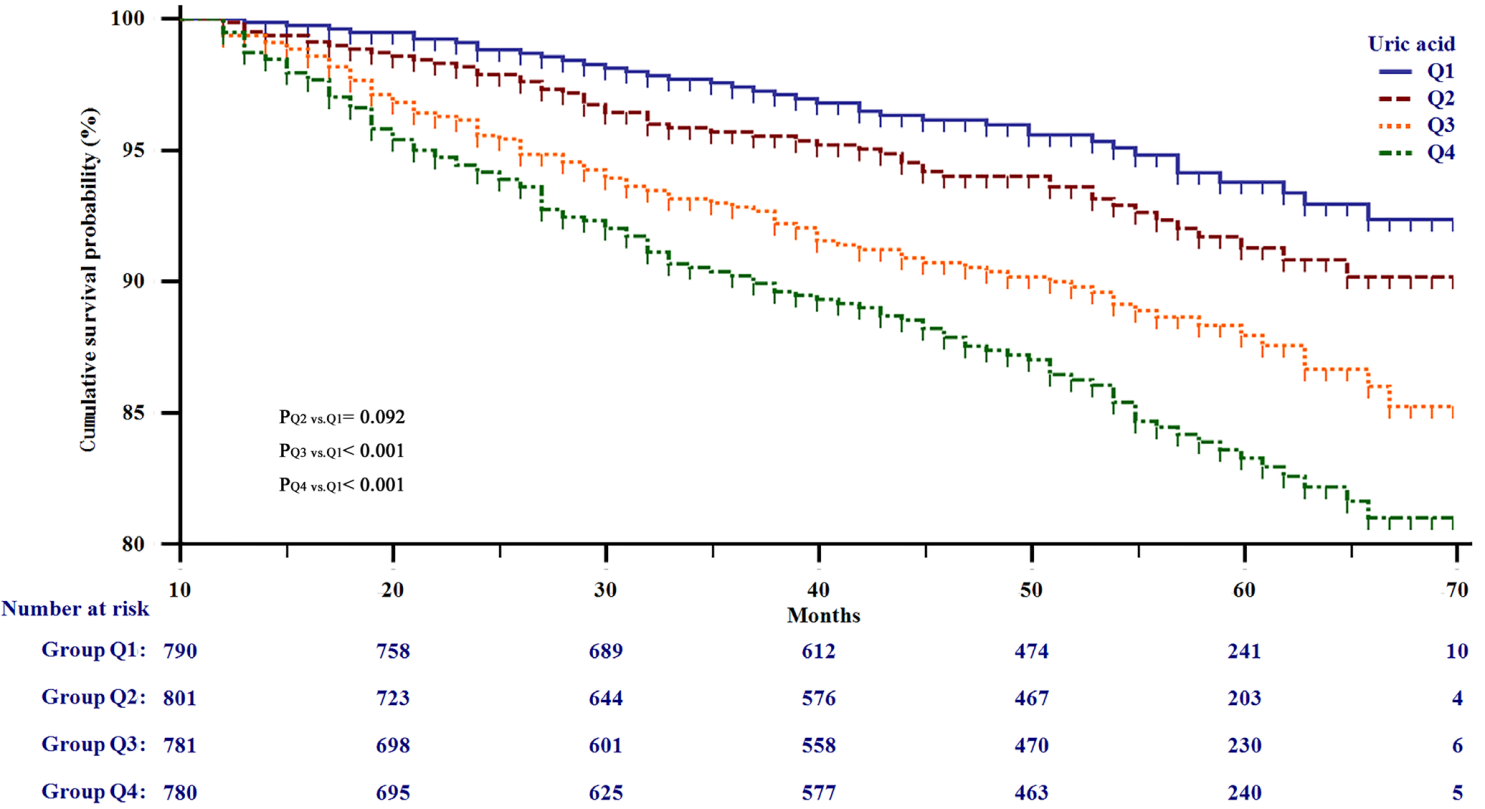
Kaplan-Meier survival analysis for all participants stratified by serum uric acid quarter

**Table 2:** Hazard Ratio (95% confidence interval) for cardio-cerebrovascular events

	Non-adjusted	P	Adjust I	P	Adjust II	P
**Q1**	**Ref**	**/**	**Ref**	**/**	**Ref**	**/**
Q2	1.42 (0.94, 2.15)	0.094	1.23 (0.82, 1.87)	0.320	1.26 (0.83, 1.92)	0.279
Q3	2.21 (1.51, 3.24)	<0.001	2.04 (1.39, 3.00)	<0.001	1.97 (1.33, 2.91)	<0.001
Q4	3.03 (2.10, 4.36)	<0.001	2.39 (1.66, 3.45)	<0.001	2.05 (1.40, 3.01)	<0.001

Adjust I model adjust for: smkoing, drinking, age, body mass index, systolic blood pressure, diastolic blood pressure.Adjust II model adjust for: smkoing, drinking, age, body mass index, systolic blood pressure, diastolic blood pressure, albumin, total bilirubin, blood urea nitrogen, creatinine, glucose, total cholesterol, triglyceride, high-density lipoprotein cholesterol, low-density lipoprotein. cholesterol, hemoglobin, platelet, white blood cell.

**Table 3:** The interaction between age, hypertension, creatinine clearance rate, glucose, triglyceride and serum uric acid for risk of cardio-cerebrovascular events

	Q1	Q2	Q3	Q4	P for interaction
Age					
< 50 y	Ref	1.31 (0.76- 2.23)	2.28 (1.39- 3.73)	2.40 (1.48- 3.88)	0.9066
≥ 50 y	Ref	1.20 (0.61- 2.37)	1.77 (0.92- 3.39)	1.99 (1.06- 3.74)	
Hypertension					
No	Ref	1.77 (0.78- 4.05)	2.39 (1.53- 7.49)	2.78 (1.76- 8.11)	0.5208
Yes	Ref	1.09 (0.67-1.78)	1.57 (0.99-2.49)	1.62 (1.03-2.55)	
Creatinine clearance rate					
≥ 80 ml/min	Ref	1.12 (0.63- 1.96)	2.49 (1.49- 4.17)	2.46 (1.48- 4.09)	0.2529
< 80 ml/min	Ref	1.33 (0.71- 2.51)	1.46 (0.79- 2.72)	1.62 (0.88- 2.98)	
Glucose					
< 5.6mmol/L	Ref	1.49 (0.88- 2.50)	2.15 (1.32- 3.51)	2.44 (1.51- 3.95)	0.3383
≥ 5.6mmol/L	Ref	0.91 (0.43- 1.92)	1.65 (0.83- 3.26)	1.82 (0.92- 3.61)	
Triglyceride					
< 1.7 mmol/L	Ref	1.41 (0.80- 2.49)	1.69 (0.95- 3.01)	2.39 (1.38- 4.14)	0.1546
≥ 1.7 mmol/L	Ref	0.91 (0.48- 1.70)	1.47 (0.85- 2.54)	1.77 (1.01- 3.10)	

Adjust for: smkoing, drinking, age, body mass index, systolic blood pressure, diastolic blood pressure, albumin, total bilirubin, blood urea nitrogen, creatinine, glucose, total cholesterol, triglyceride, high-density lipoprotein cholesterol, low-density lipoprotein. cholesterol, hemoglobin, platelet, white blood cell.

### Threshold effect analysis and conditional survival analysis

**Figure 2:**
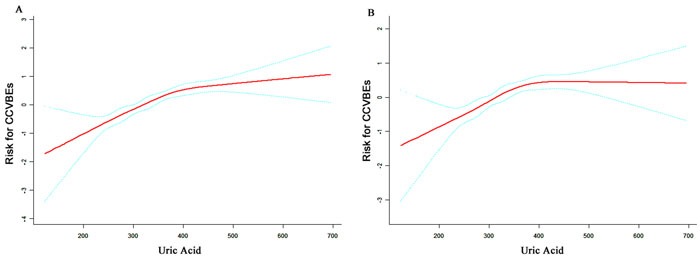
Curve-fitting between serum uric acid and risk of cardio-cerebrovascular events **A**. unadjusted; **B**. adjusted for smoking, drinking, age, body mass index, systolic blood pressure, diastolic blood pressure, albumin, total bilirubin, blood urea nitrogen, creatinine, glucose, total cholesterol, triglyceride, high-density lipoprotein cholesterol, low-density lipoprotein cholesterol, hemoglobin, platelet, white blood cell.

**Figure 3:**
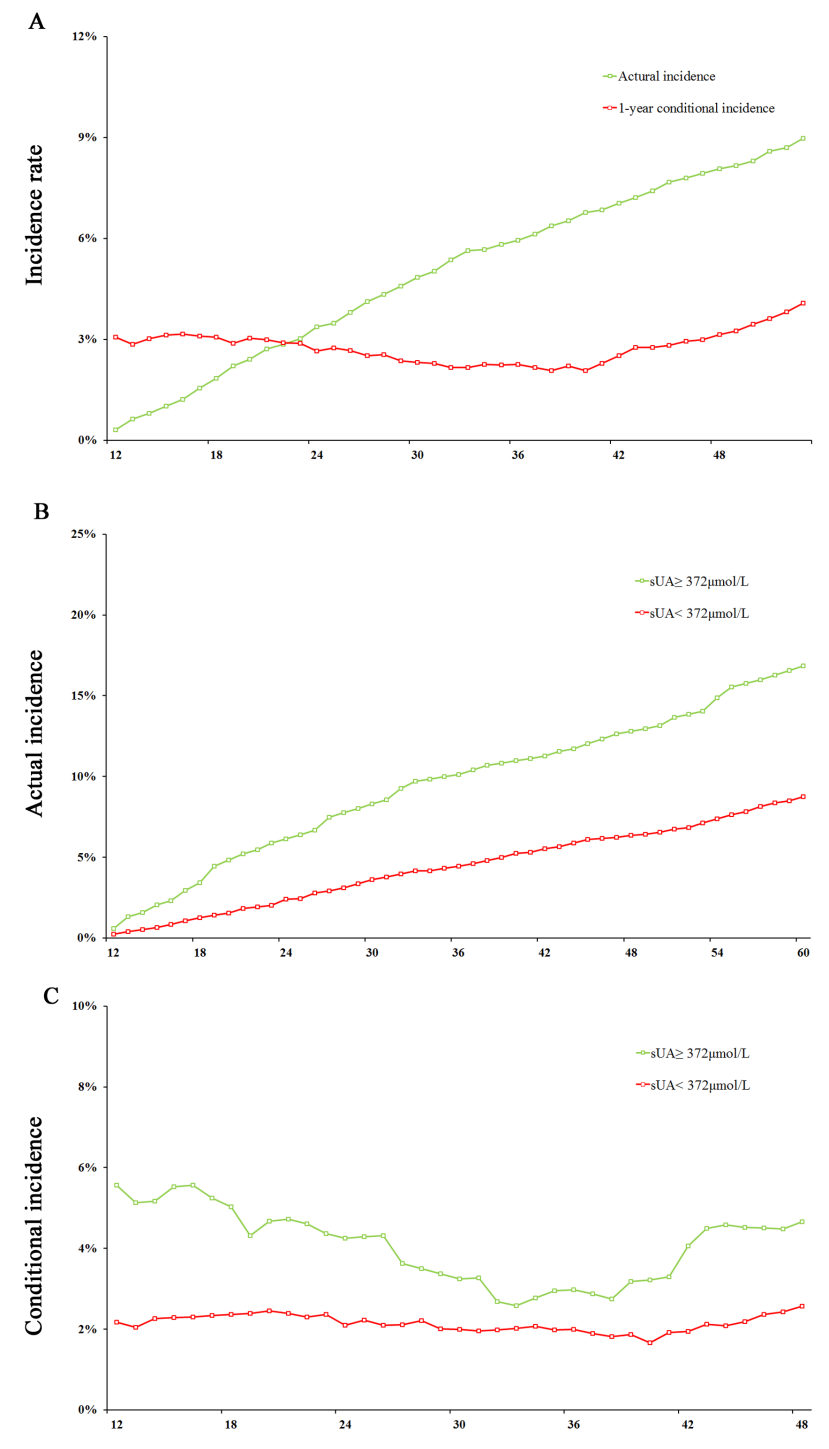
Actual incidence and 1-year conditional incidence analysis for cardio-cerebrovascular events **A**. actual incidence and conditional incidence in the total population; **B**. actual incidence in participants with high and low serum uric acid levels; **C**. conditional incidence in participants with high and low serum uric acid levels.

Cox proportional hazard regression analysis showed that the HRs of sUA did not increase linearly. Generalized additive model (GAM) was applied to explore the relationship between uric acid and risk of CCBVEs. Figure 2 shows curve-fitting for sUA and risk of CCBVEs. sUA had a positive association with risk of CCBVEs, whether the confounding variables were adjusted or not. Furthermore, we found a turning point in the curves. Threshold effect analysis was performed to confirm this point, and a point of 372 in the x-axis was observed in both Figure 2A and 2B. Figure 2A shows that the slope in sUA < 372 μmol/L was approximately 2.5 times as that in sUA < 372 μmol/L, which means that an increment of lower sUA level results in higher risk of CCBVEs. When adjusted for other confounding variables, we found that increment of sUA > 372 μmol/L do not increase the risk of CCBVEs, indicating that sUA no longer play an independent risk for CCBVEs in the high sUA level group. Subsequently, we calculated the survival rate for all participants and sub-group participants, which were stratified by the turning point (sUA = 372 μmol/L). Figure 3A shows the actual incidence (AI) increase from 0.3% in 12 months to 8.1% in 48 months. i-conditional incidence_j_ (i-CI_j_) means the incidence of CCBVEs after j months in participants who had non-CCBVEs in i months. We could observe that the CI_12_ of the total participants in each month was relatively stable, ranging from 2.1% to 4.1%. Figure 3B and 3C shows that the AI and CI_12_ in the two subgroups were stratified by the turning point. The CI_12_ in the higher sUA group was relatively lower between 33 and 39 months, but it did not show a statistically significant difference with the other months.

### Risk score by the sUA, multiple variable-based score and time-dependent ROC curves

**Figure 4:**
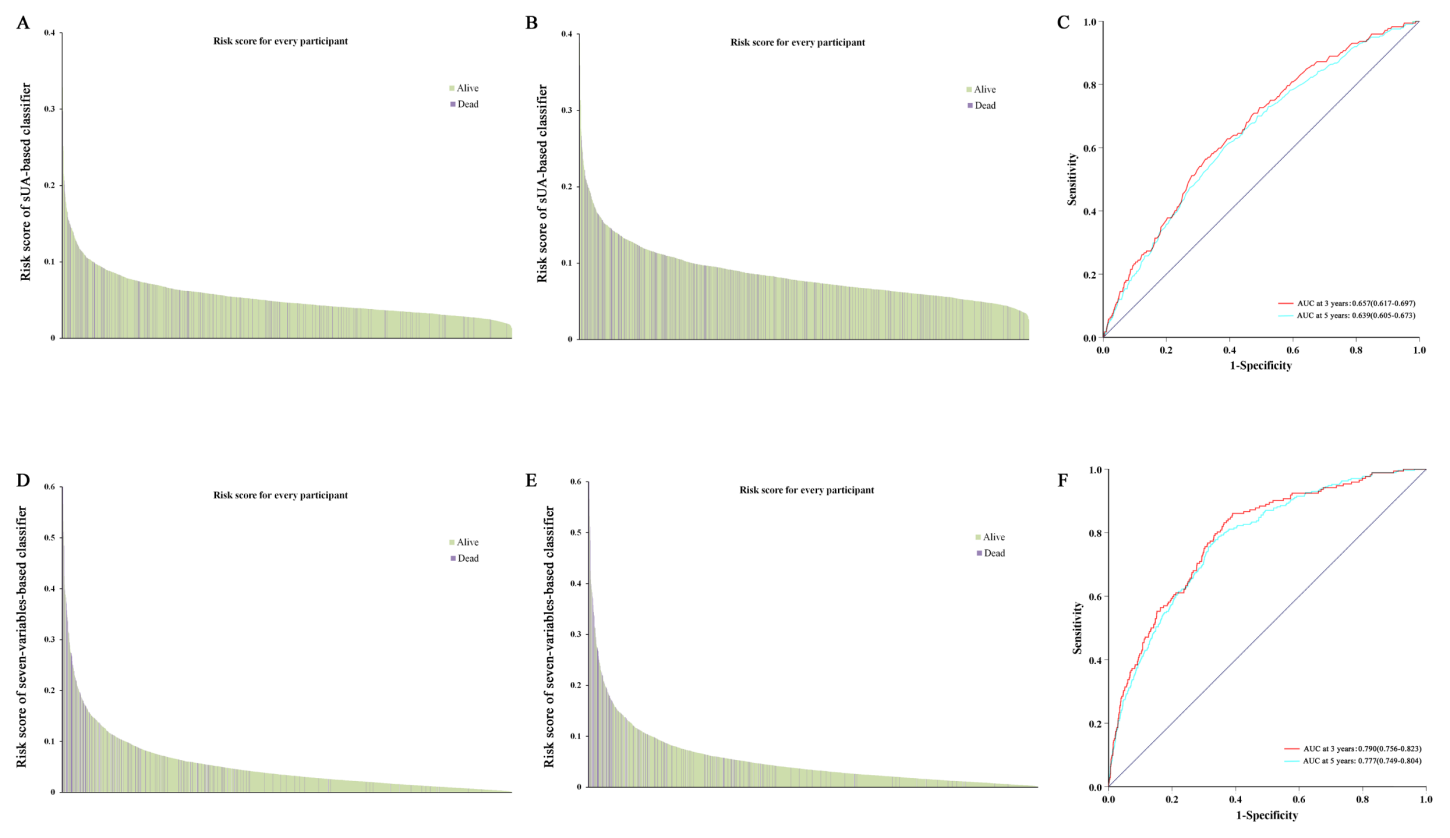
Risk score by the sUA, seven variable-based score, and time-dependent ROC curves **A**., **B**. risk of sUA-based score in 3 years and 5 years; **D**., **E**. risk of seven variable-based score in 3 years and 5 years; **C**., **F**. ROC analysis of two scores in 3 years and 5 years. ROC, receiver operator characteristic.

We performed logistics regression to calculate a multiple variable-based score at 3 years and 5 years using the most significant seven variables: age, smoking, drinking, BMI, SBP, sUA, and TG. When we assessed the distribution of risk scores and survival status, patients with lower risk scores generally had better survival than did those with higher risk scores (Figure 4A, 4B, 4D, and 4E). The distribution of multiple variable-based score in CCBVEs was more concentrated on the left than that of sUA. We assessed the prognostic accuracy of the two risk scores based on the classification with time-dependent ROC analysis at varying follow-up times (Figure 4C and 4D). The area under ROC (AUC) of sUA was 0.657 (0.617, 0.697) and 0.639 (0.605, 0.673) in 3-year and 5-year prognoses. Moreover, we can observe a higher prognostic accuracy of multiple variable-based score in 3 years and 5 years, with AUC of 0.790 (0.756-0.823) and 0.777 (0.749-0.804), respectively.

## DISCUSSION

In this longitudinal-based study in non-obese Chinese men aged 40-60 years, who were CCBVD-free at baseline, we found a strong and significant association between baseline sUA levels and risk of both coronary heart disease and stroke. These associations were attenuated by adjustment for other risk factors and were stronger in patients with lower age, blood pressure, CCr, glucose levels, or TG levels than in those with higher age, blood pressure, CCr, glucose levels, or TG levels. However, it did not show significant interactions between the five variables and sUA.

The association between sUA and cardiovascular disease was largely unnoticed until the mid-1950s and early 1960s, when it was rediscovered [14–16]. Since then, a number of epidemiologic studies have reported a relationship between sUA levels and a wide variety of vascular conditions, including hypertension [15], metabolic syndrome [17], coronary artery disease [18], cerebrovascular disease [9, 19], vascular dementia [20], and kidney disease [21]. The relationship between sUA and CCBVDs is observed not only with frank hyperuricemia, but also with normal range sUA levels [22]. In this study, we investigated the association between sUA and CCBVEs in non-obese and healthy middle-aged Chinese men. Our finding that elevated sUA level increases risk of CCBVEs is similar with previous studies on the association between sUA and CCBVDs [9, 22, 23] in healthy men, rather than women [11, 12]. In some previous studies, the association between uric acid and coronary heart disease disappeared after adjustment for potential confounders, which led to the opinion that uric acid has no role in the etiology of cardiovascular disease [11, 12]. This maybe because these studies were published during the time uric acid was regarded as a biologically inert molecule. However, recent insights in the biological effects of uric acid have falsified this view, and many epidemiological studies [10, 16, 21, 23–25], including our present study, found that uric acid plays a clear and independent role in CCBVDs. Although we can never be sure that no residual confounding remains, we do speculate that the role of uric acid in CCBVDs has been underestimated for a long time and should be reconsidered.

Several events have led to the ongoing reappraisal of the role of uric acid in CCBVDs. Some studies, such as our study, had adjusted multiple confounding risk factors and suggested that sUA may be an independent risk factor for CCBVDs [6, 9, 10, 19, 25]. However, a new view has raised that elevated sUA levels are both direct and indirect causes of CCBVDs. Recent experimental and clinical evidence supported the possibility that an elevated sUA level may lead to hypertension, renal disease, metabolic syndrome, and so on [17, 21, 26–28], which are strong independent risk factors of CCBVDs. If uric acid caused hypertension, metabolic syndrome, renal disease, then evaluated uric acid level might not be independent of these diseases when as a risk factor for CCBVDs. Thus, a better understanding of the biologic functions of uric acid related to CCBVDs is needed.

We demonstrated that sUA is a stable risk factor for CCBVEs. More attention should be paid to this easily available and inexpensive serum marker in clinics. Furthermore, finding a turning point in curve-fitting is interesting. Figure 2 shows that the increment of lower sUA level results in higher CCBVEs risk than that of higher sUA levels. The benefit of hypouricemic would be outstanding until the sUA level decreases to a specific value (turning point in this study).

The strengths of this study include its longitudinal population-based design, adjustment for confounding risk factors, and exclusion of CCBVDs and cancer at baseline. The limitations of this study require further comment. First, our findings may be specific to Chinese Han and may exhibit inherent bias because our study is localized in Wuhan, and the sample size is relative small. Second, many factors may influence the outcome of CCBVEs because it is a complex disease, and we could not consider all the confounding factors, such as lifestyle and dietary factors, in our study. Further studies including more complete personal information are needed to explore the causal relationship between uric acid and CCBVEs. Third, defining the relationship between uric acid and CCBVEs is less evident because of the observational nature of this study. Studies with treatment would be needed in the future.

In conclusion, sUA is independently associated with CCBVEs and acts as an indicator of CCBVEs when adjusted for the confounding risk factors.

## MATERIALS AND METHODS

### Approval of the ethics committee

This is a prospective observational study conducted from June 2007 to June 2010 in the health examination center of Tongji Hospital, Tongji Medical College, Huazhong University of Science and Technology. The study design and protocol were approved by the Institutional Ethical Review Committee of Tongji Hospital. The study subjects were informed of the possibility of using the data obtained for academic purpose. Confidentiality was assured to all participants, and data used for this study were stripped of personally identifiable information. They were also informed regarding their right to withdraw their consent at any point, without any consequence.

### Patient selection criteria and follow-up

The study participants were all male aged 40-60 years, with BMI < 25kg/m^2^, and who had a regular annual health examination in Tongji Hospital. We initially included 4702 participants from June 2007 to June 2010. They were selected based on the following exclusion criteria: 1) those who visited only once (935 subjects); 2) those who had CCBVEs within a year (38 subjects); and 3) history of myocardial infarction, stroke, chronic renal failure, liver disease, and cancer (312 subjects). Finally, the remaining 3152 subjects were enrolled as the study cohort. In the present study, we defined CCBVEs as major vascular-related events with high incidence: myocardial infarction, angina angioplasty, coronary artery bypass surgery, transient ischemic attack, or stroke. Arrhythmia was not taken into account. The participants’ CCBVEs were self-reported by the participants at the annual health examination and validated by medical certificate, which were obtained from their electronic medical record (EMR). If participants did not arrive at the stipulated time, the participants are called to ensure completeness and timeliness of follow-up.

### Data collection

Clinical examination and data recording were conducted in the morning after an overnight fast, and the subjects were instructed to refrain from performing exercises a day before their examination. Medical history and health habit inventory were conducted by trained medical staff by using a standardized procedure.

The data on demographic information, such as age, sex, previous stroke history, smoking, alcohol drinking, and so on were collected for the first time during health examination. Subjects who smoked at least 10 cigarettes per day for 6 months or more were considered as smokers. Drinking was defined as alcohol consumption > 140 g/week. The heights and body weights of study subjects were recorded to the nearest 0.5 cm or 0.5 kg, respectively. BMI, used as an index of body fat, was calculated as the ratio of weight (kg) to height (m^2^). Blood pressure (BP) measurements were performed with participants in the seated position and after a quiet resting period of 10 min. BP was measured in the right arm with a noninvasive automated sphygmomanometer (OMRON, Japan). Hypertension was diagnosed when a patient was administered medications for hypertension, or had SBP ≥ 140 mmHg and/or DBP ≥ 90 mmHg after the 5-min rest.

Fasting blood samples were collected from each subject in the antecubital vein and were used for the analysis of biochemical measurements of the serum samples without freezing. Blood samplings were performed to test albumin, total bilirubin, glucose, BUN, creatinine, UA, TC, TG, HDL-C, LDL-C, hemoglobin, platelet count, and WBC. The Ccr was calculated by using the formula: Ccr = (140 − age) × weight (kg)/ [72 × Scr (mg/dL)].

### Statistical analysis

Continuous variables of normal and skewed distributions were expressed as mean standard deviation (SD) and median (interquartile range), respectively. Categorical values were expressed by absolute and relative frequencies. Follow-up time was calculated by the date difference between the first and last health examinations. We compared two groups by using the t test for continuous variables and χ^2^ test for categorical variables. First, the participants were classified into four groups by using the sUA quarter: Q1 < 279 μmol/L, 279 μmol/L ≤ Q2 < 323.5 μmol/L, 323.5μmol/L≤ Q3 < 375 μmol/L, and 375 ≤ Q4μmol/L. The HRs and 95% confidence intervals (CIs) for CCVBEs were calculated after adjusting for known confounding variables across each quartile of sUA concentration by using multivariate logistic regression analysis. Kaplan-Meier analysis and the log-rank test were applied to compare incidence the risk of CCBVEs among different uric acid groups. We applied the GAM to explore the relationship between uric acid and risk of CCBVDs, and the threshold effect analysis to find the turning point. We used the Cox regression model to perform the multivariable analysis, and the cox regression coefficients to generate a new prognosis score. All the statistical analyses were performed with R software version 3.0.1 (R Development Core Team 2013). *P* < 0.05 (two-sided) was considered as statistical significance.

## SUPPLEMENTARY MATERIALS FIGURES AND TABLES


