# Supplementary material for: Serum uric acid level as a cardio-cerebrovascular event risk factor in middle-aged and non-obese Chinese men
Source: Oncotarget. 2017 Mar 4;8(15):24110–8. doi: 10.18632/oncotarget.15902 (PMC5421831; doi:10.18632/oncotarget.15902)
Supplement: Supplementary file 1 [file oncotarget-08-24110-s001.pdf]

Serum uric acid level as a cardio-cerebrovascular event risk factor in middle-aged and non-obese Chinese men

Supplementary Material

Supplementary Table 1. Number of each event in different sUA groups

	Q1	Q2	Q3	Q4
MI	13	18	29	36
Angina angioplasty	17	25	39	62
CABG	3	2	3	3
Stroke	10	16	18	31
TIA	7	6	8	15
Total hospitalization	35	50	77	101

Q1 < 279 µmol/L, 279 µmol/L ≤ Q2 < 323.5 µmol/L, 323.5µmol/L≤ Q3 <375 µmol/L, 375 ≤ Q4µmol/L
